# Structure-specific recognition protein-1 (SSRP1) is an elongated homodimer that binds histones

**DOI:** 10.1074/jbc.RA117.000994

**Published:** 2018-05-15

**Authors:** Gabriele Marcianò, Stefano Da Vela, Giancarlo Tria, Dmitri I. Svergun, Olwyn Byron, Danny T. Huang

**Affiliations:** From the ‡ Cancer Research UK Beatson Institute, Garscube Estate, Switchback Road, and the Institute of Cancer Sciences, University of Glasgow, Glasgow G61 1BD, Scotland, United Kingdom,; the § European Molecular Biology Laboratory, Hamburg Outstation, EMBL ℅ DESY, Notkestrasse 85, 22607 Hamburg, Germany, and; the ¶ School of Life Sciences, University of Glasgow, Glasgow G12 8QQ, United Kingdom

**Keywords:** analytical ultracentrifugation, small-angle X-ray scattering (SAXS), histone chaperone, oligomerization, histone, H2A–H2B, H3–H4, homodimer, SSRP1

## Abstract

The histone chaperone complex facilitates chromatin transcription (FACT) plays important roles in DNA repair, replication, and transcription. In the formation of this complex, structure-specific recognition protein-1 (SSRP1) heterodimerizes with suppressor of Ty 16 (SPT16). SSRP1 also has SPT16-independent functions, but how SSRP1 functions alone remains elusive. Here, using analytical ultracentrifugation (AUC) and small-angle X-ray scattering (SAXS) techniques, we characterized human SSRP1 and that from the amoeba *Dictyostelium discoideum* and show that both orthologs form an elongated homodimer in solution. We found that substitutions in the SSRP1 pleckstrin homology domain known to bind SPT16 also disrupt SSRP1 homodimerization. Moreover, AUC and SAXS analyses revealed that SSRP1 homodimerization and heterodimerization with SPT16 (resulting in FACT) involve the same SSRP1 surface, namely the PH2 region, and that the FACT complex contains only one molecule of SSRP1. These observations suggest that SSRP1 homo- and heterodimerization might be mutually exclusive. Moreover, isothermal titration calorimetry analyses disclosed that SSRP1 binds both histones H2A–H2B and H3–H4 and that disruption of SSRP1 homodimerization decreases its histone-binding affinity. Together, our results provide evidence for regulation of SSRP1 by homodimerization and suggest a potential role for homodimerization in facilitating SPT16-independent functions of SSRP1.

## Introduction

In eukaryotes DNA is packaged into nucleosomes, which consist of 147-bp fragments of DNA wrapped around a histone octamer comprising two H2A–H2B heterodimers and one H3–H4 heterotetramer (1). Nucleosomes represent a barrier for DNA replication, repair, and transcription machinery. Hence, they need to be reorganized to allow access to DNA. Nucleosome assembly is a process that occurs during transcription and DNA replication. It can be described as a two-step process where DNA is initially contacted by a histone H3–H4 tetramer and subsequently two histone H2A–H2B dimers (2–4). This two-step process is carried out by histone chaperones. There are two classes of histone chaperones grouped according to their mechanism of action. The first class uses ATP hydrolysis to move or modify histone structure along the DNA, and the second class reorganizes nucleosomes without ATP hydrolysis (5–7). Facilitates chromatin transcription (FACT)^5^ belongs to the latter group (5–8).

FACT is a heterodimeric complex consisting of structure-specific recognition protein-1 (SSRP1) and suppressor of Ty 16 (SPT16) (9, 10). It plays important roles in DNA replication, transcription, and DNA repair by remodeling chromatin structure, although the mechanism remains elusive (11–14). Early *in vitro* studies demonstrated that FACT displaces the histone H2A–H2B dimer from the nucleosome (9, 15, 16), but Xin *et al.* (16) showed that FACT also increases nuclease access to DNA without H2A–H2B eviction. Based on these data, two models were proposed to explain FACT's mechanism of action, the “dimer eviction and insertion” model and the “accessibility and tethering” model. In the first model, FACT actively removes histones H2A–H2B from the nucleosome to enable DNA accessibility, and in the second model FACT tethers to the nucleosome components without H2A–H2B eviction and destabilizes the nucleosome conformation (17).

**Figure 1.**
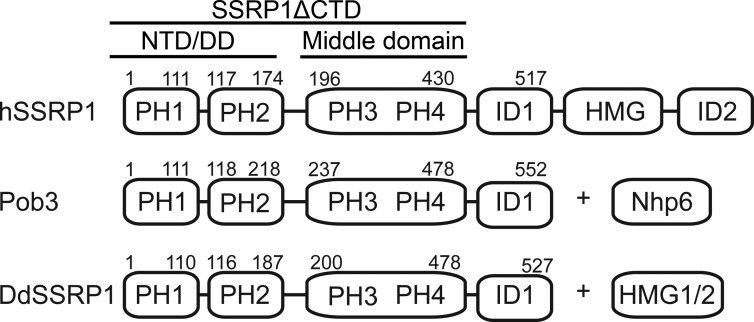
**Schematic representation of hSSRP1 (*top*), Pob3 (*middle*), and DdSSRP1 (*bottom*).** *Nhp6,* nonhistone protein 6.

Several studies showed that FACT binds to all components of the nucleosome, including histones H2A–H2B and H3–H4, histone N-terminal tails, and DNA via domains within FACT subunits SPT16 and SSRP1 (9, 18–27). SPT16 consists of an N-terminal domain, a dimerizing domain, and a middle domain followed by an intrinsic disordered region at the C terminus (13, 24, 28). The SPT16 N-terminal domain adopts an aminopeptidase-like structure that binds histones H3–H4 (21, 23, 26). The dimerizing domain assumes a pleckstrin homology (PH)-like fold, important for SPT16–SSRP1 heterodimerization (20). The SPT16 middle domain adopts a double PH domain structure similar to that of Rtt106 and the SSRP1 middle domain and binds histone H2A–H2B and H3–H4 (20, 27). The C-terminal acidic region of SPT16 binds a hydrophobic pocket on H2B (29). SSRP1 consists of two N-terminal PH domains (PH1–PH2), a middle domain (MD) comprising a double PH domain (PH3–PH4), and a high-mobility group (HMG) domain flanked by two intrinsic disordered regions (ID1 and ID2) (Fig. 1). The homolog of SSRP1 in yeast is the Pob3–Nhp6 complex; Pob3 comprises two N-terminal PH domains and a double PH domain, whereas Nhp6 comprises a HMG-1 domain. Studies showed that SSRP1 binds histone H3–H4 (19); the PH2 domain interacts with the dimerizing domain of SPT16 (20); and SSRP1 MD and HMG domains interact with DNA (30–32). Interestingly, the C-terminal acidic regions of Pob3 and SPT16 bind overlapping sites on H2B suggesting that FACT might bind to the two symmetry–related H2A–H2B dimers in the nucleosome (29). In this manner, FACT would break up DNA–histone interactions to promote nucleosome reorganization.

In cells, the bulk of cellular SSRP1 is found in complex with SPT16 (9, 33). Thus, SSRP1 and SPT16 functions are tightly associated with the roles played by FACT in chromatin remodeling. However, several studies have shown that in addition to its FACT function, SSRP1 exhibits SPT16-independent functions. SSRP1 has been shown to associate with transcription factors such as the serum-response factor and p63 to regulate their activity (34, 35). Indeed, SSRP1 and SPT16 knockdown transcriptome analyses revealed a distinct set of genes that are regulated by SSRP1 (36). Furthermore, SSRP1 has been shown to facilitate microtubule growth in mitosis (37) and to regulate DNA demethylation in *Arabidopsis* (38). It remains unclear how SSRP1 behaves independently of SPT16. To further elucidate the function of SSRP1, we characterized its solution structure using analytical ultracentrifugation (AUC) and small-angle X-ray scattering (SAXS), and we showed that SSRP1 self-associates to generate an elongated homodimer. Mutational analyses revealed that SSRP1 PH2 and PH3 domains are essential for homodimerization. Moreover, SSRP1 homodimerization and SPT16 interaction utilize the same SSRP1 surface suggesting that both events might be mutually exclusive. Finally, we showed that optimal histone binding requires SSRP1 homodimerization. Collectively, our results provide a hint for how SSRP1 could function independently of SPT16.

## Results

### SSRP1 is a homodimer

**Figure 2.**
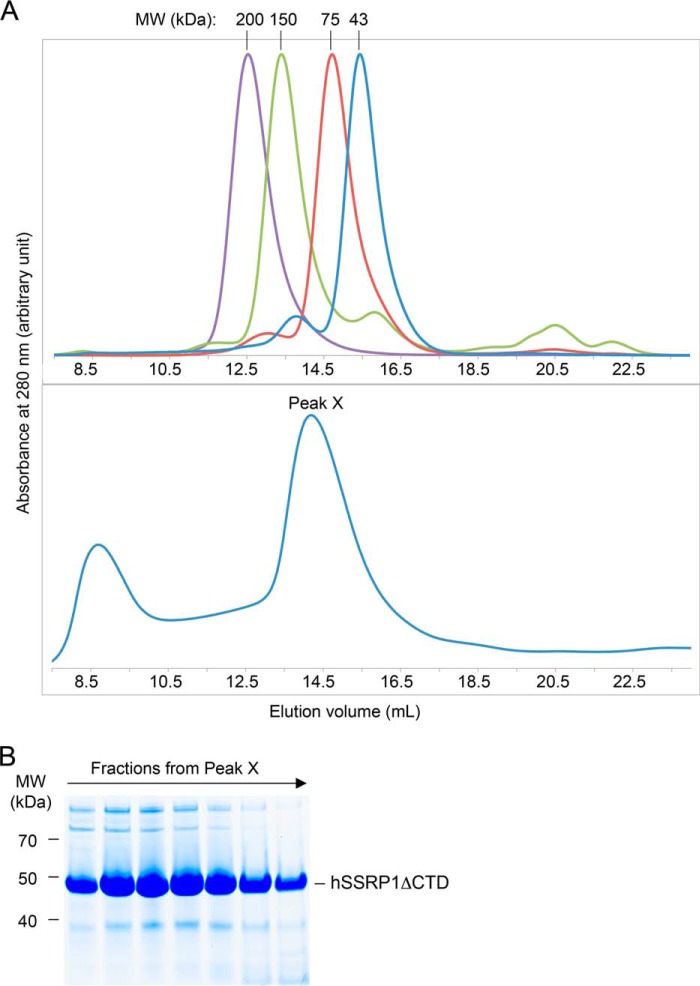
**Analysis of hSSRP1 by gel-filtration chromatography.** *A,* elution profile from an SD200 10/30 size-exclusion chromatography column of protein standards: β-amylase (200 kDa), alcohol dehydrogenase (150 kDa), ovalbumin (43 kDa) and conalbumin (75 kDa) (*top panel*) and hSSRP1ΔCTD. Peak X contains hSSRP1ΔCTD (*bottom panel*). *B,* SDS-PAGE of fractions from peak X in *A, bottom panel*.

To better characterize SSRP1 structure and function, we expressed and purified human SSRP1 lacking the C-terminal ID regions and the HMG domain (residues 1–433; hSSRP1ΔCTD) for biochemical and biophysical analysis (Fig. 1). hSSRP1ΔCTD, with a predicted molecular mass of 49.8 kDa, eluted between the 150- and 75-kDa molecular mass markers in size-exclusion chromatography, suggesting it might form an oligomer or adopt an elongated conformation (Fig. 2).

**Figure 3.**
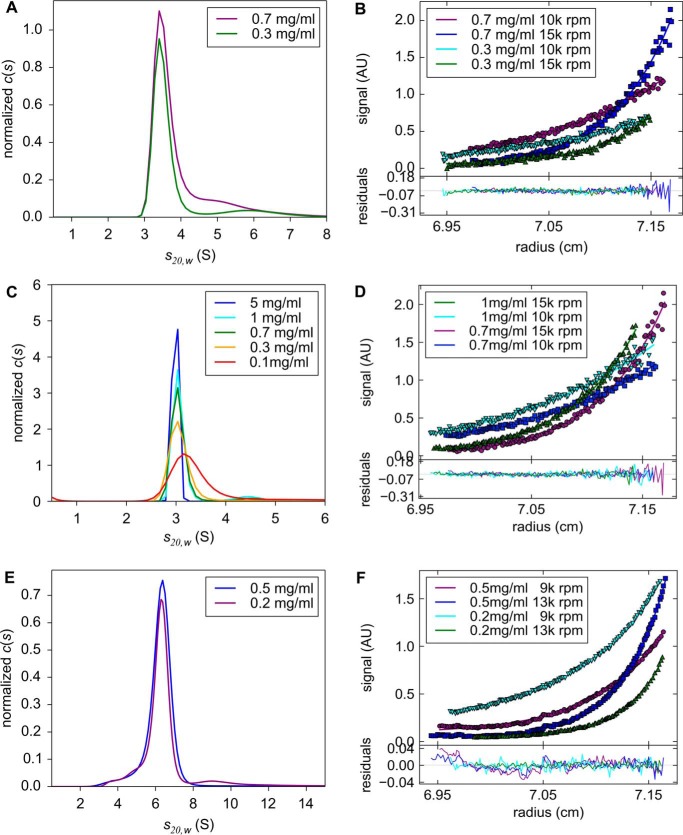
**AUC analyses of SSRP1.** *A, c*(*s*) distribution derived from SV of hSSRP1ΔCTD at various protein concentrations. *B,* SE analyses of hSSRP1ΔCTD. Data are globally fitted with a single species model. *C, c*(*s*) distributions derived from SV of DdSSRP1ΔCTD revealing similar sedimentation coefficients as hSSRP1 ΔCTD. *D,* SE analyses of DdSSRP1ΔCTD. Data are globally fitted with a single species model. *E,* c(s) distribution derived from SV of DdFACT showing that the protein is monodispersed with the same sedimentation coefficient at 0.5 and 0.2 mg/ml. *F,* SE analyses of DdFACT. Data are globally fitted with a single species model. Images were made using the software GUSSI (65).

To determine the oligomeric state and molecular mass of hSSRP1ΔCTD in solution, AUC was performed. Sedimentation velocity (SV) data showed that hSSRP1ΔCTD has an infinite dilution sedimentation coefficient *s*_20, *w*_^0^ of 3.32 ± 0.21 S. The frictional ratio (*f/f*_0_) provides information about the shape of the protein: globular proteins have a ratio between 1.2 and 1.3; elongated, asymmetric, or glycosylated proteins lie between 1.5 and 1.8, and unfolded or linear chains have a much higher ratio (39, 40). hSSRP1ΔCTD gave a value of 1.63, suggesting that it is elongated in solution. Sedimentation equilibrium (SE) data globally fitted with a single species model yielded a molecular mass of 91.8 ± 3.7 kDa, which is similar to the predicted molecular mass of a homodimer (99,615 Da; Fig. 3, *A* and *B*).

Next, we assessed whether SSRP1 homodimerization is conserved among different species. *Dictyostelium discoideum* SSRP1 (DdSSRP1) adopts a similar domain structure as Pob3 containing N-terminal PH domains followed by a C-terminal ID region (Fig. 1). We expressed and purified DdSSRP1 lacking the C-terminal ID region (residues 1–478; DdSSRP1ΔCTD). Sedimentation velocity and equilibrium data confirmed that DdSSRP1ΔCTD has an *s*_20, *w*_^0^ (3.23 ± 0.1 S) similar to that of hSSRP1ΔCTD and a molecular mass of 104.8 ± 22.7 kDa (Fig. 3, *C* and *D*; predicted molecular mass of a dimer is 108,466 Da), demonstrating that homodimerization is conserved.

### Second PH domain and middle domain are required for homodimerization

SSRP1 consists of two N-terminal PH domains and a middle domain, which is a double PH domain. For simplicity, we designate these PH domains as PH1–4 (Fig. 1). The crystal structure of the SSRP1–SPT16 heterodimer reveals that SSRP1 PH2 is responsible for heterodimerization with the dimerizing domain of SPT16 (20). Because the dimerizing domain of SPT16 adopts a PH-like fold similar to those found in SSRP1, we hypothesized that homodimerization of SSRP1 may involve its PH2 domain. DdSSRP1 was used in all subsequent analyses as it was more stable when mutations were introduced. We introduced six DdSSRP1 mutations (6mut: C112A, N113A, W114A, F135R, M172R, and M175R) along the SSRP1 PH2–SPT16 dimerizing domain–binding interface and assessed whether this mutant has a defect in homodimerization. AUC analysis showed that DdSSRP1ΔCTD_6mut is a monomer in solution at all tested concentrations in the range of 0.2–60 μm (Table 1), suggesting that SSRP1 PH2 is required for homodimerization. To further probe the mechanism of homodimerization, we generated DdSSRP1 containing only PH1 and PH2 domains (DdSSRP1_NTD; Fig. 1). AUC analysis showed that DdSSRP1_NTD is a monomer at all tested concentrations in the range of 2.4 to 304 μm (Table 1), suggesting that the PH1 and PH2 domains are insufficient for homodimerization and require the presence of PH3–PH4.

**Table 1:** **Sedimentation equilibrium analysis of DdSSRP1 constructs**

Protein	Mass	σ	Mass expected
	*Da*		*Da*
DdSSRP1ΔCTD_6mut	58,417.56	5346.65	54,102.16
DdSSRP1_NTD	20,249.15	2808.91	20,481.08
DdSSRP1ΔCTD Q306K	54,827.28	4626.82	54,223.44

A single point mutation, Q308K, in yeast Pob3 causes defects in transcription and replication (24). This mutation has no effect on Pob3 MD structure but alters the surface charge in that region (24). We next assessed whether the corresponding substitution, Q306K, in DdSSRP1ΔCTD has an effect on homodimerization. We performed AUC analysis on DdSSRP1ΔCTD Q306K and found that it is a monomer at all tested concentrations in the range of 1–41 μm (Table 1). Together, these results suggest that the SSRP1 homodimerization interface may involve both the PH2 and PH3 domains.

### FACT is a heterodimer of SSRP1 and SPT16

Given that SSRP1 homodimerization and SSRP1–SPT16 heterodimerization require the same SSRP1 PH2 surface, we investigated the oligomeric state of FACT complex. To address this question, we performed AUC on the DdFACT complex. DdFACT was purified as described under “Experimental procedures.” SV data showed that DdFACT is homogeneous in solution with an *s*_20, *w*_^0^ = 6.09 ± 0.52 S, and a frictional ratio of 1.51, suggesting that the complex is elongated. SE data gave a molecular mass of 162.6 ± 18.8 kDa, which agrees with the sequence molecular mass of 162,856 Da (Fig. 3, *E* and *F*), indicating that DdFACT does not oligomerize. Our results are consistent with the earlier finding showing that yeast FACT is an elongated heterodimer (33). Together, the data suggest that SSRP1 homodimerization and SSRP1–SPT16 heterodimerization cannot occur simultaneously.

### SAXS analysis

**Figure 4.**
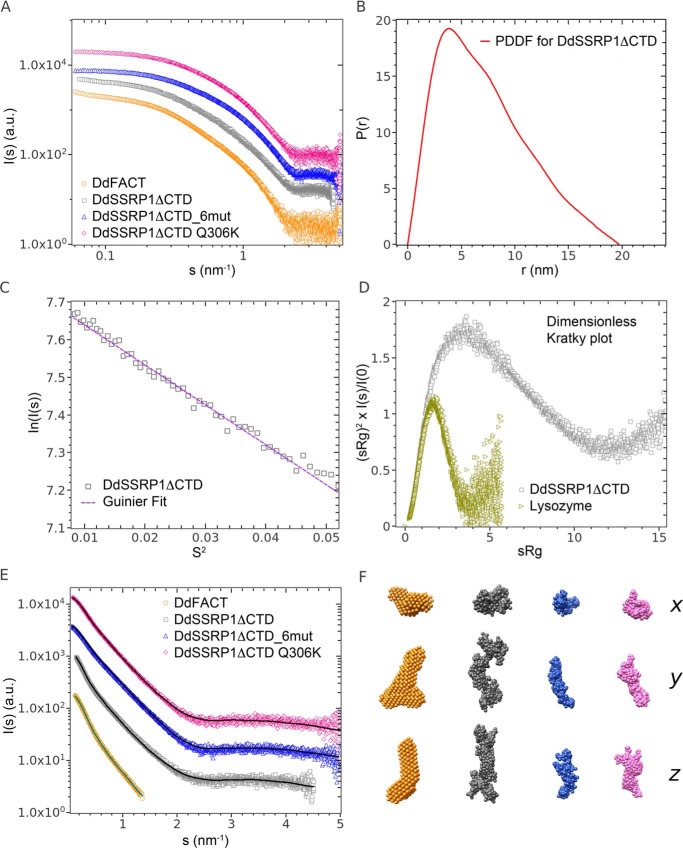
**SAXS analyses of DdSSRP1ΔCTD.** *A,* double logarithmic plot showing the merged SAXS profiles used to extract overall parameters and for DAMMIF modeling of the FACT heterodimer, arbitrarily displaced in the vertical direction for display. *B,* DdSSRP1ΔCTD WT pair distance distribution function *P*(*r*) (*PDDF*). *C,* Guinier plot; *D,* dimensionless Kratky plot with a globular protein for comparison of DdSSRP1ΔCTD WT. *E,* comparison of the SAXS data used for structural modeling (*symbols*) with the calculated scattering curves from DAMMIF and GasborMX (*solid lines*). For the FACT complex, the DAMMIF modeling employed the merged curve up to 1.4 nm^−1^. For DdSSRP1ΔCTD and mutants, the GasborMX modeling employed the SAXS profiles at the highest concentration (WT 9 mg/ml, 6mut 12.2 mg/ml, and Q306K 10.9 mg/ml). The pairs of fits are shifted by about 1 order of magnitude along the logarithmic axis for clarity. *F,* final averaged (“filtered”) *ab initio* model of FACT and three representative GasborMX models of DdSSRP1ΔCTD WT, 6mut, and Q306K in three orthogonal views along the *x*, *y*, and *z* axes. From *left to the right*: DdFACT heterodimer (*orange*), DdSSRP1ΔCTD WT homodimer (*gray*), monomer DdSSRP1ΔCTD_6mut (*blue*), and monomer DdSSRP1ΔCTD Q306K (*pink*).

Solutions of DdSSRP1ΔCTD, of the heterodimeric FACT complex, and of the DdSSRP1ΔCTD mutants, 6mut and Q306K, were studied with synchrotron SAXS to gain insight into overall SSRP1 structure and oligomeric state. The results are presented in Fig. 4.

Guinier analysis (see “Experimental procedures”) was performed on the scattering profiles, yielding the radii of gyration (*R_g_*) and the molecular mass of the proteins, with the latter based on the scattering intensity extrapolated to zero angle (*I*(0)). The *R_g_* and mass values as functions of the protein concentration are shown in Figs. S1 and S2, respectively. These overall parameters suggest that the heterodimeric FACT complex and DdSSRP1ΔCTD have similar sizes, with estimated *R_g_* values between 5.5 and 6.0 nm. The estimated mass of the FACT complex (130–150 kDa) is relatively close to the mass calculated from its primary sequence (162.856 kDa). In contrast, the mass for DdSSRP1ΔCTD (90–110 kDa), points to its homodimerization, as the mass calculated from the sequence is 54.233 kDa. These overall parameters thus further corroborate the results from AUC.

The SSRP1ΔCTD mutants, 6mut and Q306K, are apparently smaller than DdSSRP1ΔCTD, with the *R_g_* around 4.5 nm, and their scattering profiles are similar to each other (Fig. 4*A*). The estimated mass values are in the range 50–80 kDa, which tend toward the sequence-calculated values (54.102 kDa for 6mut and 54.233 kDa for Q306K). Therefore, the overall parameters from SAXS suggest that the mutants are predominantly monomeric. However, the apparent mass and *R_g_* values grew with concentration pointing to possible dimerization effects.

Table 2 reports the overall parameters obtained after appropriate merging of lower and higher concentrations of SAXS data, including *R_g_*, the maximum particle size *D*_max_, the Porod (excluded) volumes, and the mass. The relative differences between the proteins measured are confirmed, as well as the indication of a dimeric assembly for the WT DdSSRP1ΔCTD. The merged SAXS data for DdSSRP1ΔCTD are further analyzed in Fig. 4, *B–D*. Both the pair distance distribution function (*P*(*r*)) and the dimensionless Kratky plot suggest an elongated structure for the homodimer, deviating from the overall fold of typical globular proteins. The good linearity of the Guinier region confirms the successful removal of low-*s* concentration dependences.

**Table 2:**
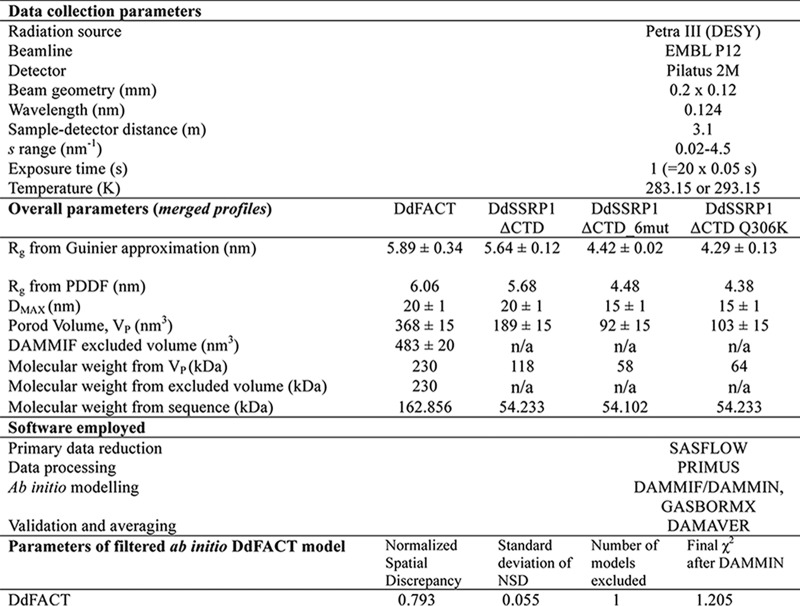
**Data collection and SAXS-derived parameters**

The merged profile for the FACT complex was further utilized to obtain the *ab initio* model of its low-resolution shape using the program DAMMIF (41) (see “Experimental procedures”). For the WT DdSSRP1ΔCTD and its two mutants showing concentration-dependent dimerization, the program GasborMX was employed to generate the shapes of monomers and dimers while simultaneously fitting multiple scattering data sets at different concentrations (see “Experimental procedures”). The resulting *ab initio* models and fits to the experimental SAXS are displayed in Fig. 4, *E* and *F*, and the concentration dependence of the monomer and dimer fractions for DdSSRP1ΔCTD WT, 6mut, and Q306K is given in Fig. S3.

From the above *ab initio* modeling, FACT heterodimer has an elongated asymmetric shape. Both DdSSRP1ΔCTD and its mutants demonstrate a concentration-dependent homodimerization. However, only WT DdSSRP1ΔCTD reveals significant amounts of dimers at low protein concentration. The mutants are mostly dissociated into monomers at concentrations below 1 mg/ml, with a slight tendency for increased dimerization at higher concentrations (Fig. S3). Given these results, at the (low) concentrations used for AUC, an essentially monomeric state is expected. All the DdSSRP1ΔCTD homodimers are elongated (Fig. S4), with the length comparable with the FACT heterodimer but with a smaller cross-section. Interestingly, the dimers seem to adopt an extended, V-shaped conformation in solution.

### Homodimerization plays a role in histone binding

**Figure 5.**
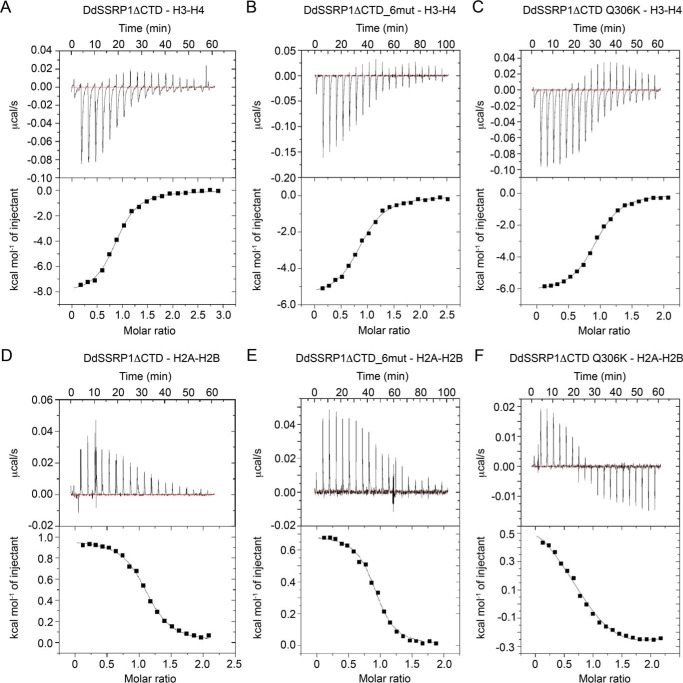
**ITC profiles of the interaction between DdSSRP1 variants and histones.** *A,* DdSSRP1ΔCTD-H3–H4 binding profile. *B,* DdSSRP1ΔCTD_6mut-H3–H4 binding profile. *C,* DdSSRP1ΔCTD Q306K-H3–H4 binding profile. *D,* DdSSRP1ΔCTD-H2A–H2B binding profile. *E,* DdSSRP1ΔCTD_6mut-H2A–H2B binding profile. *F,* DdSSRP1ΔCTD Q306K-H2A–H2B binding profile. Raw data (*top panel*) and integrated data (*bottom panel*) are shown. The data shown are representative of two independent experiments except *C, E,* and *F* that are representative of one experiment.

SSRP1 has been shown to have SPT16-independent function in gene transcription as knockdown of SPT16 and SSRP1 in human nonsmall cell lung carcinoma cells revealed a subset of genes that are regulated by SSRP1 independent of SPT16 (36). Previous studies showed that SSRP1 binds nucleosomes with high affinity and has a preference for binding histone H3–H4 (19). We hypothesized that SSRP1 homodimerization contributes to histone binding. Currently, we do not know how SSRP1 homodimerizes, and therefore it was difficult to generate an SSRP1 variant that solely affects homodimerization. Given that DdSSRP1ΔCTD Q306K and DdSSRP1ΔCTD_6mut are monomeric in solution at low concentrations, we tested their effects on histone binding. We performed ITC analyses to assess how DdSSRP1ΔCTD, DdSSRP1ΔCTD Q306K, and DdSSRP1ΔCTD_6mut bind histones. DdSSRP1ΔCTD bound histone H3–H4 with a *K_d_* of 0.57 ± 0.05 μm (Fig. 5*A* and Table 3), which is similar to the binding affinity observed between human SSRP1 and *Xenopus laevis* histone H3–H4 (19). Interestingly, DdSSRP1ΔCTD_6mut and DdSSRP1ΔCTD Q306K bound histone H3–H4 with a *K_d_* of 3.0 ± 0.2 μm and 1.37 ± 0.07 μm, respectively (Fig. 5, *B* and *C*, and Table 3). Thus, disruption of homodimerization via Q306K substitution or mutations in the PH2 domain decreased histone H3–H4 binding affinity by 5- or 2.5-fold, respectively. We also tested SSRP1 binding affinity with histone H2A–H2B. DdSSRP1ΔCTD bound histone H2A–H2B with a *K_d_* of 0.82 ± 0.05 μm (Fig. 5*D* and Table 3), whereas DdSSRP1ΔCTD_6mut exhibited a slightly weaker binding affinity with a *K_d_* of 1.57 ± 0.14 μm (Fig. 5*E* and Table 3). The ITC profile for DdSSRP1ΔCTD Q306K binding is biphasic (Fig. 5*F*) suggesting that the Q306K substitution likely alters the surface charge thereby initiating a nonspecific H2A–H2B binding. Together, our ITC analyses show that SSRP1 homodimerization contributes to histone binding.

**Table 3:** **ITC analysis of DdSSRP1 constructs against histone H2A–H2B or H3–H4**

SSRP1–histone binding	*K_d_*
	μ*m*
DdSSRP1ΔCTD–H2A–H2B	0.82 ± 0.05
DdSSRP1ΔCTD_6mut–H2A-2B	1.57 ± 0.14
DdSSRP1ΔCTD–H3–H4	0.57 ± 0.05
DdSSRP1ΔCTD_6mut–H3–H4	3.0 ± 0.2
DdSSRP1ΔCTD Q306K–H3–H4	1.37 ± 0.07
DdSSRP1_FL–H2A–H2B	0.31 ± 0.03
DdSSRP1_ID1–H2A–H2B	0.19 ± 0.03

### DdSSRP1 ID1 region binds histone H2A–H2B

**Figure 6.**
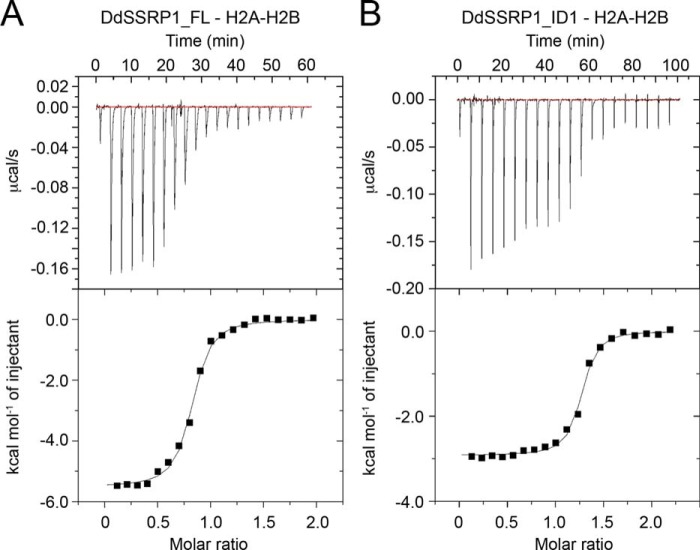
**ITC profiles of the interaction between DdSSRP1 ID1 region and histone H2A–H2B.** *A,* DdSSRP1_FL-H2A–H2B binding profile. *B,* DdSSRP1_ID1-H2A–H2B binding profile. Raw data (*top panel*) and integrated data (*bottom panel*) are shown. The data shown are representative of one independent experiment.

Recent studies on Pob3 showed that its acidic ID1 region binds directly to histone H2A–H2B (29, 42). To investigate whether the ID1 region of DdSSRP1 also binds histone H2A–H2B, we performed ITC analyses using full-length DdSSRP1 (DdSSRP1_FL) and DdSSRP1 containing only the ID1 region (residues 479–527; DdSSRP1_ID1). Titration of DdSSRP1_FL against histone H2A–H2B generated an exothermic thermogram with a *K_d_* of 0.31 ± 0.03 μm (Table 3) that is different from the endothermic thermogram generated by titration of DdSSRP1ΔCTD against histone H2A–H2B (compare Figs. 5*D* and 6*A*), suggesting that the ID1 region likely binds histone H2A–H2B, and the interaction is exothermic. Indeed, DdSSRP1_ID1 bound histone H2A–H2B with a *K_d_* of 0.19 ± 0.03 μm, and the thermogram exhibited an exothermic reaction (Fig. 6*B* and Table 3). These results showed that the ID1 region of DdSSRP1 binds to histone H2A–H2B.

## Discussion

Little is known about how SSRP1 behaves independent of SPT16; our present work reveals a role of SSRP1 homodimerization. We show that both human and *D. discoideum* SSRP1 self-associate to form an elongated homodimer in solution using both AUC and SAXS analyses. Homodimerization likely involves both PH2 and PH3 domains as mutations within these two domains yield monomeric protein. Notably, the PH2 surface that interacts with SPT16 also participates in homodimerization, suggesting that both binding events might be mutually exclusive. Indeed, we show that the DdFACT complex does not oligomerize, consistent with an earlier study on yeast FACT (33). Furthermore, we show that disruption of SSRP1 homodimerization decreases the binding affinity of SSRP1 for histones H2A–H2B and H3–H4, highlighting a role for homodimerization in the function of SSRP1.

Homodimerization has been observed in other histone chaperones such as Rtt106 (43, 44), yeast nucleosome assembly protein 1 (Nap1) (45), and vacuolar protein sorting 75 (Vps75) (46, 47). These histone chaperones harbor an N-terminal helical domain that is involved in dimerization. Homodimerization plays an important role in their functions as follows. Rtt106 homodimerization is essential for its association with histone H3–H4 tetramer and its function in transcriptional silencing (44); Nap1 homodimerization enables it to interact with two molecules of histones H2A–H2B or H3–H4 (48); and Vps75 homodimerization is involved in binding and activating the catalytic activity of histone acetyltransferase Rtt109 (47, 49). SSRP1 lacks a defined homodimerization domain. We show that the PH1–PH2 domain is a monomer, and structural studies of MD from human and Pob3 also reveal a monomer (24, 30). Homodimerization seems to be achieved via multiple surfaces involving both PH2 and PH3 domains. The exact molecular details of homodimerization will require future studies. Here, we show that monomeric forms of SSRP1 mutants are able to bind both histones H2A–H2B and H3–H4 with low micromolar affinity, but the presence of a homodimer further enhances the binding affinity. Given that SSRP1 interacts with other proteins independent of SPT16, future studies will be required to elucidate whether homodimerization has a role in other SSRP1 functions.

**Figure 7.**
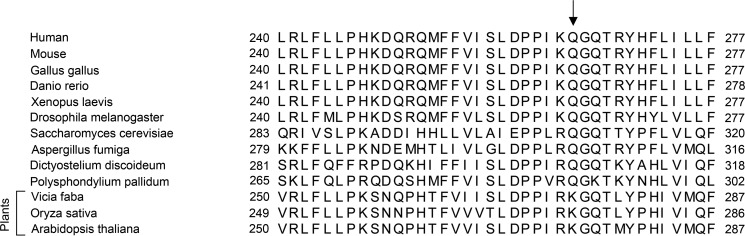
**Sequence alignment of SSRP1 from different species.** Sequences near DdSSRP1 Gln-306 are shown. *Arrow* indicates Gln-306 in DdSSRP1. Residue numbers are indicated.

In our study, we show that both human and *D. discoideum* SSRP1 are homodimers, but whether homodimerization is conserved in other species remains unclear. Sequence analysis of SSRP1 from plants, fungi, and mammals shows that SSRP1 comprising N-terminal PH domains and the ID1 region is conserved. Closer inspection reveals that Gln-306 (in *D. discoideum*) is conserved from fungi to mammals, but the corresponding residue in plants is a lysine (Fig. 7). In our study, DdSSRP1ΔCTD Q306K is predominantly a monomer at low protein concentrations, but it has the tendency to dimerize at high protein concentrations. Thus, this criterion alone is not sufficient to predict its oligomeric state. Future studies are required to assess the oligomeric state of SSRP1 in other species.

We showed that SSRP1 homodimerization and SPT16 heterodimerization utilize the same SSRP1's PH2 surface, and the FACT complex contains one molecule of SSRP1. These data suggest that both events could compete for the same pool of SSRP1. This raises the question of how SSRP1 oligomerization is regulated. In HeLa and yeast cells the FACT complex concentration is in the range of 0.1–0.5 μm, suggesting that the bulk of SSRP1 is associated with SPT16 (9, 33). Our SAXS analyses showed that SSRP1 homodimerization is concentration-dependent where the monomeric fraction increases at lower protein concentrations (Fig. S3). At 1 mg/ml (∼18 μm), DdSSRP1ΔCTD is predominantly a dimer but with a small fraction in the monomeric state. Thus, it seems that at a low protein concentration, SSRP1 has a stronger preference to form a complex with SPT16, and the SSRP1 homodimer would be present when SSRP1 is in excess of SPT16 and at higher protein concentration. Future studies are required to understand how SSRP1 homodimerization is regulated.

Studies have shown that human SSRP1 binds nucleosome, histone H3–H4, and DNA (19, 30), and Pob3 binds histone H3–H4 (50). In this study, we show that DdSSRP1 binds histones H2A–H2B and H3–H4 via different mechanisms. DdSSRP1ΔCTD bind histone H3–H4 with a binding affinity similar to that of human SSRP1 (19). Interestingly, both PH1–4 domains (DdSSRP1ΔCTD) and the C-terminal ID1 region (DdSSRP1_ID1) bind histone H2A–H2B independently. A very recent study showed that the C-terminal acidic ID1 region of Pob3 containing a (D/E)*XX*Φ (where Φ is Phe or Tyr, and *X* is any residue; DEDF in Pob3) motif binds H2B (29), and the reported binding affinity is similar to the binding affinity of DdSSRP1_ID1 for histone H2A–H2B observed here. Furthermore, DdSSRP1 harbors a DDDY motif in the ID1 region, suggesting a similar mode of H2A–H2B binding. It is unclear how DdSSRP1 PH1–4 domains bind H2A–H2B, and whether the binding site is distinct or overlaps with the ID1 region requires future investigation. It is noteworthy that the C-terminal acid region of yeast Spt16 contains an EVSEY motif that binds to the same H2B pocket as the acidic ID1 region of Pob3, and their H2A–H2B binding mode is incompatible with H2A–H2B–DNA interaction in the nucleosome structure (29). Thus, it was proposed that the SPT16 and SSRP1 C-terminal acidic regions of FACT would bind to two symmetry-related H2A–H2B dimers to compete with DNA thereby promoting nucleosome reorganization. The distance between the two H2B-binding pockets in the nucleosome structure is about 60 Å, and the nucleosome has a dimension of 100 × 100 × 60 Å. With the extended V-shaped conformation and *D*_max_ of 200 Å (Table 2), DdSSRP1 homodimer containing two C-terminal acidic ID1 regions could potentially perform a similar function. SSRP1 has the characteristic of a histone chaperone because it binds all components of the nucleosome. Future studies are required to elucidate how SSRP1 binds histones and to determine whether it could modulate nucleosome assembly independent of SPT16.

## Experimental procedures

### Protein expression and purification

Human and *D. discoideum* (Dd) SSRP1 variants were cloned into RSF_Duet vector (Novagen) and expressed with an N-terminal hexahistidine (His_6_) tag followed by a TEV cleavage site. All proteins were expressed in *Escherichia coli* BL21 (DE3) Gold (Stratagene) and purified by using Ni-NTA affinity chromatography followed by TEV treatment to remove the His_6_ tag. The cleaved proteins were further purified by anion-exchange and size-exclusion chromatography. Histone core octamers were purified from chicken blood, as described previously (51), and separated into H2A–H2B and H3–H4 using cation-exchange chromatography. DdFACT complex was obtained by coexpressing His_6_-tagged DdSSRP1 residues 1–478 (in RSF_Duet vector; see above) and GSH–*S*-transferase (GST)-tagged DdSpt16 residues 1–955 (in pGEX4T1 vector containing an N-terminal GST tag followed by a TEV cleavage site) in *E. coli* BL21 (DE3) Gold. The DdFACT complex was purified by using Ni-NTA and GSH-Sepharose affinity chromatography followed TEV treatment to remove the tags. The cleaved complex was further purified by anion-exchange and size-exclusion chromatography. All proteins where purified in 25 mm Tris-HCl, pH 7.6, 150 mm NaCl, 1 mm DTT. Protein concentration was determined at 280 nm using a calculated extinction coefficient.

### Analytical ultracentrifugation

Purified SSRP1 or DdFACT was dialyzed against buffer containing 25 mm Tris-HCl, pH 7.6, 200 mm NaCl, 2 mm tris(2-carboxyethyl)phosphine and concentrated. AUC was carried out in a Beckman Coulter Optima XL-I analytical ultracentrifuge (Palo Alto, CA). SV experiments were performed at 4 °C at a rotor speed of 49,000 rpm. Samples (360 μl) at various concentrations, were loaded into double-sector centerpieces. Data were acquired every 7 min with interference and absorbance optics and were subsequently analyzed using size-distribution (c(*s*) *versus s*) analysis in SEDFIT (52). Partial specific volume, buffer density, and viscosity at 4 and 20 °C were calculated using SEDNTERP (53). SE experiments were carried out with the same range of protein concentrations using 90 μl of sample with different rotor speeds according to the predicted molecular mass. Scans were taken every 3 h until equilibrium was confirmed using WinMATCH (Jeffrey Lary, University of Connecticut, Storrs, CT). SE data were analyzed using SEDPHAT (54) and fitted with a species analysis model.

### Small-angle X-ray scattering

Synchrotron X-ray solution–scattering data were collected at the EMBL P12 beamline (PETRA III, DESY, Hamburg, Germany) (55) using a robotic sample changer (56). Initially, the data were reduced and processed using an automatic pipeline of software developed at EMBL Hamburg, Germany (57). SSRP1 was prepared in 25 mm Tris-HCl, pH 7.6, 150 mm NaCl, 1 mm DTT to obtain concentration series in the range 0.5 and 12.2 mg ml^−1^. SAXS data were recorded at 10 or 20 °C using a PILATUS 2M pixel detector (DECTRIS, Baden, Switzerland) at a sample-detector distance of 3.1 m and a wavelength of 0.124 nm. This configuration covers a range of momentum transfer of 0.12 < s < 5.0 nm^−1^ (s = 4π sin(θ)/λ, where 2θ is the scattering angle). The software PRIMUS (58, 59) was used for data processing. The intensity calibration was performed using the scattering from BSA at a known concentration as a secondary standard. The forward scattering *I*(0) and *R_g_* values were determined using the Guinier approximation assuming that at very small angles (*s* < 1.3 *R_g_*) the intensity is represented as *I*(s) = *I*(0)·exp(−(*s R_g_*)^2^/3). To take into account the concentration-dependent effects in the scattering curves, merged scattering profiles were obtained by combining the smaller angle portion (up to about 1.1–1.3 nm^−1^) of SAXS data collected at low concentrations with the higher angle portion (starting from about 0.6–0.9 nm^−1^) of high concentrations of SAXS data. The pair-distance distribution function *P*(*r*), from which the maximum particle dimension (*D*_max_) and *R_g_* were estimated, was computed using GNOM (60). The molecular masses were derived from the following: 1) extrapolation to zero scattering angle on absolute scale; 2) the excluded volume of the hydrated particle using the Porod invariant (59), and 3) the excluded volumes of *ab initio* bead models. The latter were generated from the low resolution data (*s* <1.4 nm^−1^) for the FACT complex by repeating 20 DAMMIF runs (61) without symmetry (P1) or anisometry and refined with DAMMIN (41). Both algorithms construct bead models yielding a scattering profile with the lowest possible discrepancy (χ) from the experimental data while keeping beads interconnected and the model compact. Twenty independent *ab initio* reconstructions were performed and averaged using DAMAVER (62), which also provides a normalized spatial discrepancy as a measure of similarity among different reconstructions. SAXS methods and results are summarized in Table 2.

The homodimerization behavior of SSRP1 WT and mutants, as well as the structure of the dimers, were analyzed by GasborMX (59). The program simultaneously fits SAXS profiles of the concentration series with a linear combination of the scattering intensities of monomer and dimer. The monomer structure is represented as a collection of dummy residues, and the dimer is generated in a P2 symmetry. To better model the dimers, prolate anisometry was imposed, with the dyadic axis of the symmetric dimer being transverse to the major axis of the model. For the concentration series treated, the modeling was repeated 10 times, and the best fitting models were selected. Alignment of the *ab initio* models for depiction and comparison purposes was performed using SUPCOMB (63). The *ab initio* models shown in Fig. 4 and in the supporting information were rendered using the UCSF Chimera package (64).

### Isothermal titration calorimetry

SSRP1 binding to histone H2A–H2B or H3–H4 was determined at 25 °C by using a MicroCal iTC200 microcalorimeter (Malvern Instruments Ltd., UK). All proteins were buffer-exchanged into 20 mm HEPES, pH 7.5, 200 mm NaCl, 1 mm DTT. Histone H2A–H2B or H3–H4 was loaded into the cell at a concentration of 20–30 μm, and SSRP1 variants were loaded into the syringe at a concentration 10 times higher than that in the cell. 20 injections (2 μl each) were added every 180 s to the cell. For control experiments, buffer or SSRP1 was injected into the cell containing histone or buffer, respectively. ITC data were generated by subtracting the raw data from the control experiment and were analyzed using Origin software (version 7, OriginLab).

## Author contributions

G. M. data curation; G. M. and D. T. H. formal analysis; G. M. and D. T. H. supervision; G. M. and D. T. H. investigation; G. M. and D. T. H. writing-original draft; G. M. and D. T. H. project administration; G. M., S. D. V., D. I. S., and D. T. H. writing-review and editing; O. B. methodology; D. T. H. conceptualization; D. T. H. funding acquisition; S. D. V., G. T., and D. I. S. performed and analyzed SAXS experiment.

## Supplementary Material

Supporting Information

## Footnotes

5 The abbreviations used are:

FACT: facilitates chromatin transcription
FL: full length
PH: pleckstrin homology
TEV: tobacco etch virus
ΔCTD: ΔC-terminal domain
ITC: isothermal titration calorimetry
AUC: analytical ultracentrifugation
SAXS: small-angle X-ray scattering
MD: middle domain
HMG: high-mobility group
SV: sedimentation velocity
SE: sedimentation equilibrium
Ni-NTA: nickel-nitrilotriacetic acid.
