# Supplementary material for: Structure-specific recognition protein-1 (SSRP1) is an elongated homodimer that binds histones
Source: J Biol Chem. 2018 May 15;293(26):10071–83. doi: 10.1074/jbc.RA117.000994 (PMC6028955; doi:10.1074/jbc.RA117.000994)
Supplement: Supporting Information [file supp_293_26_10071__index.html]

Structure specific recognition protein-1 (SSRP1) is an elongated homodimer that binds histones — SSRP1 homodimerization has a role in histone binding — Structure-specific recognition protein-1 (SSRP1) is an elongated homodimer that binds histones — SSRP1 homodimerization has a role in histone binding — Supporting Information 

# Structure-specific recognition protein-1 (SSRP1) is an elongated homodimer that binds histones

## Supporting Information

- Structure specific recognition protein-1 (SSRP1) is an elongated homodimer that binds histones - In this supporting information, we show plots of the overall parameters derived by SAXS as a function of concentration for all the proteins measured, the dependency of the fraction of monomer and dimer on the concentration of SSRP1 and its mutants and the structures of the dimeric form of the mutants.
