# Supplementary material for: Structure-specific recognition protein-1 (SSRP1) is an elongated homodimer that binds histones
Source: J Biol Chem. 2018 May 15;293(26):10071–83. doi: 10.1074/jbc.RA117.000994 (PMC6028955; doi:10.1074/jbc.RA117.000994)
Supplement: Supporting Information [file supp_RA117.000994_133962_2_supp_137605_p8qvv0.pdf]

SUPPORTING INFORMATION

Structure specific recognition protein-1 (SSRP1) is an elongated homodimer that binds histones

Gabriele Marcianò[§], Stefano Da Vela[¶], Giancarlo Tria[¶], Dmitri I. Svergun[¶], Olwyn Byron[†] and Danny T. Huang[§]

[§] Cancer Research UK Beatson Institute, Garscube Estate, Switchback Road, Glasgow G61 1BD, United Kingdom and Institute of Cancer Sciences, University of Glasgow, Glasgow, G61 1BD, United Kingdom. [¶]European Molecular Biology Laboratory, Hamburg Outstation, EMBL c/o DESY, Notkestrasse 85, 22607 Hamburg, Germany. [†]School of Life Sciences, University of Glasgow, Glasgow G12 8QQ, United Kingdom.

In this supporting information, we show plots of the overall parameters derived by SAXS as a function of concentration for all the proteins measured, the dependency of the fraction of monomer and dimer on the concentration of SSRP1 and its mutants and the structures of the dimeric form of the mutants.

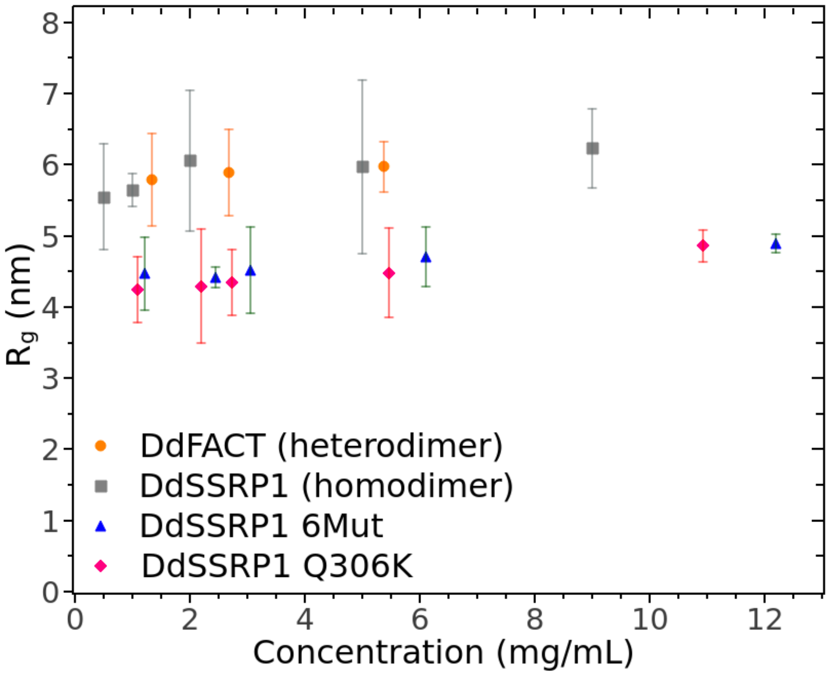

Figure S1. Radius of gyration (R_g) from Guinier analysis. The radius of gyration is slightly affected by the concentration. The DdSSRP1 dimers have R_g of approximately 5.5 to 6.0 nm and the DdSSRP1 mutants have R_g around 4.5 nm. The error bars represent the AUTORG error (1)(sum of the standard deviation from the Guinier fit and the standard deviation of the R_gs from all the AUTORG consistent data intervals).

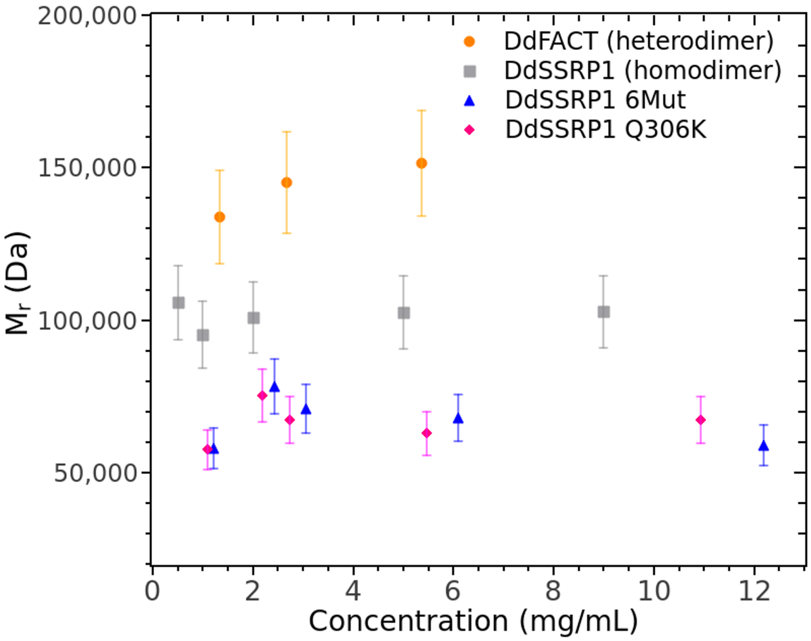

Figure S2. Molecular weight (M_r) obtained at different concentrations from the forward scattering, I(0). I(0) was extracted from Guinier analysis on SAXS profiles scaled to absolute intensity Error bars on M_r reflect the error propagation of the error on I(0) and uncertainty in the protein concentration.

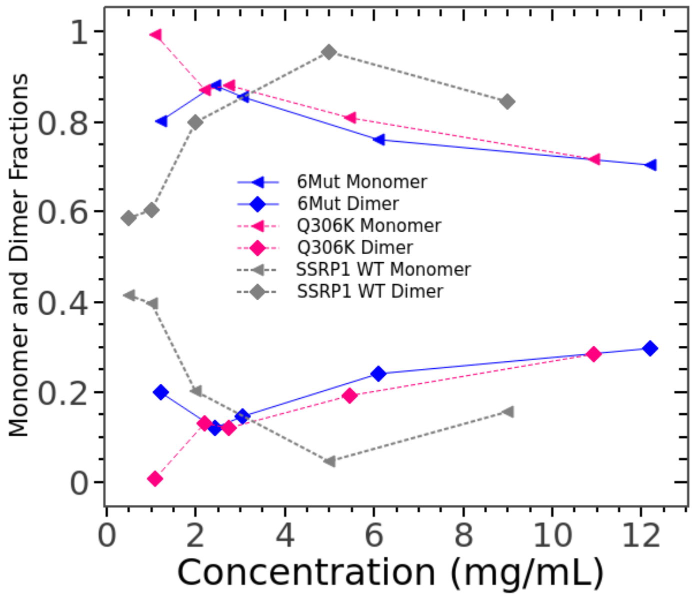

Figure S3. Fraction of monomer and dimer for the species undergoing dimerization, as a function of concentration, as extracted by the GasborMX analysis. Diamond symbols correspond to the dimer, triangles to the monomer. While the DdSSRP1 mutants are predominantly monomeric and show a slight increase in dimer fraction with the concentration, the Wild-type features a significant excess of dimer even at low concentrations.

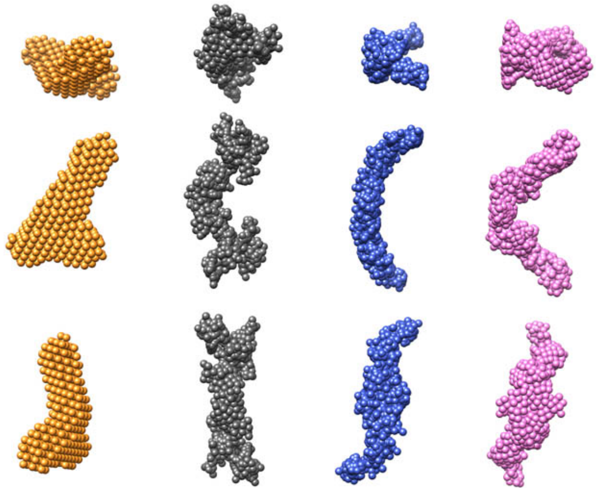

Figure S4. Comparison of FACT and DdSSRP1ΔCTD, 6mut and Q306K in three orthogonal views. From left to the right: DdFACT heterodimer (orange), DdSSRP1ΔCTD wild-type homodimer (gray), dimer of DdSSRP1ΔCTD 6mut (blue), dimer of DdSSRP1ΔCTD Q306K (pink).

1. Petoukhov, M. V., Konarev, P. V., Kikhney, A. G., and Svergun, D. I. (2007) ATSAS 2.1 - towards automated and web-supported small-angle scattering data analysis. *Journal of Applied Crystallography* **40**, s223-s228
